# Bibliometric analysis of worldwide research trends on breast cancer about inflammation

**DOI:** 10.3389/fonc.2023.1166690

**Published:** 2023-04-19

**Authors:** Guangran Meng, Huilin Xu, Shengtao Yang, Feixiang Chen, Wenyuan Wang, Furong Hu, Gang Zheng, Yixin Guo

**Affiliations:** ^1^ Department of General Surgery, The Fifth Hospital of Wuhan, Wuhan, Hubei, China; ^2^ Department of Oncology, Fifth Hospital of Wuhan, Wuhan, Hubei, China; ^3^ Department of Anesthesiology, The Fifth Hospital of Wuhan, Wuhan, Hubei, China; ^4^ Cardiac Rehabilitation Center, Wuhan Asia Heart Hospital, Wuhan, Hubei, China

**Keywords:** Breast cancer, inflammation, VOSviewer, bibliometrics, hotspots

## Abstract

**Background:**

The most prevalent cancer and the second-leading cause of cancer-related mortality in women is breast cancer. Growing interest has been shown in recent years in learning more about the processes behind the development of breast cancer. It has been shown that persistent inflammation may play a significant role in the advancement of breast cancer. However, a comprehensive and objective analysis on the state of inflammation in breast cancer research is still lacking. This study was aim to undertake a bibliometric analysis of breast cancer research associated with inflammation between 2013 and 2022 in order to identify the trends, dynamics, and scientific outputs in the field.

**Methods:**

From 2013 to 2022, original and review publications on breast cancer and inflammation-associated research were retrieved from the Web of Science Core Collection (WOSCC) database. To examine the position of yearly publications, journals, nations, institutions, and authors, we employed two bibliometric tools (CiteSpace and VOSviewer). After that, by examining keyword visualization and keyword bursts, we determined the hot research fields related to inflammation in breast cancer.

**Results:**

we discovered 6902 publications regarding inflammation in breast cancer by using our retrieval approach. In terms of the number of publications, The United States ranked first in the global study, followed by China and Italy. In terms of institutions, the University of Texas System, UT MD Anderson Cancer Center, and University of California System are in the top 3 for the quantity of publications published. The most popular journal for this field research is “CANCERS.” Ueno NT, Woodward WA, Cristofanilli M, and others have made significant contributions to the understanding of inflammation in breast cancer. In the end, we conducted a biclustering analysis on keywords and discovered three clusters that represent research hotspots.

**Conclusion:**

According to the global trend, the research output of inflammation in breast cancer is increasing. The information provided in this article, including the cooperation network information of authors, nations, journals, and institutions, may help researchers to better understand hotspots and developing patterns in this discipline. At present, the focus of study gradually shifts from “phenotype study” to “therapeutic research”. It is recommended to pay attention to the latest hot spots, such as targeted therapy, antimicrobial activity and nanoparticle.

## Introduction

Prior to 2020, lung cancer had the highest incidence rate among women worldwide. However, according to the most recent worldwide cancer data in 2020, breast cancer has officially overtaken lung cancer and taken the position as the most prevalent malignancy afflicting women globally (1). The clinical prognosis for breast cancer patients has markedly improved over time because to advancements in diagnostic and treatment approaches, with a 5-year survival rate of more than 90% for early breast cancer (2). However, patients treatment failure and death are often caused by metastatic breast cancer and distant metastases. In order to combat this medical issue, it is vital to comprehend the mechanisms of tumor growth and distant metastasis. The role of inflammation in the development and spread of breast cancer has been confirmed (3–6). Inflammation is involved in the development of breast cancer in several ways, including promoting cell proliferation, differentiation, tumor metastasis, angiogenesis, and regulating the inflammatory microenvironment in associated tissues (7). Multiple studies described the use of the inflammatory infiltrate in breast cancer as a predictive marker in the 1990s. The term “inflammatory infiltration” was initially used by Aaltomaa et al. to describe it as a prognostic sign in breast cancer. They discovered that tumor diameter, the presence of axillary lymph nodes, and lymphocyte infiltration were all positively associated (8). In 1995, Stewart et colleagues examined over 25,000 immune suppressed women over the course of 1 to 11 years and discovered a much lower incidence of breast cancer diagnoses than they had anticipated. This hypothesis suggests that immunological stimulation is crucial for the development and spread of breast cancer (9). IL-6 signaling has been shown to play an important role in tumor proliferation, migration, and adhesion (10). Adipose-derived IL-6 has been reported to promote breast cancer metastasis by inducing PLOD2 expression through activation of the JAK/STAT3 and PI3K/AKT signaling pathways (11, 12). In a recent study on triple-negative breast cancers, inhibiting the expression of IL-6 significantly inhibited the proliferation of cancer cells *in vitro* and *in vivo* (13, 14). This evidence indicates that inflammatory factors play an important role in breast cancer. According to a research, angiogenesis and macrophage counts are significantly positively correlated in breast tumors, with more macrophages present in highly vascular tumors (15). Additionally, it has been suggested that inflammation-related programmed cell death such as apoptosis, pyroptosis and necroptosis plays a significant role in the development of breast cancer (16). Although the amount of published literature on the relationship between inflammation and breast cancer has grown quickly, accurate and useful data on topics like the quantity of pertinent publications, relevant countries, institutions, journals, authors, and frequently used keywords in research on inflammation and breast cancer are still lacking.

A popular method for evaluating academic papers is bibliometrics. Research data is presently widely available due to the creation of scientific databases like Web of Science (WOS), which helps the advancement of bibliometric research (17). Bibliometrics is a comprehensive approach that combines quantitative and qualitative analyses to reveal a variety of features of publications, including identifying the nations, journals, authors and institutions that contribute to a particular research area, displaying frequently cited studies and keywords, and establishing the collaboration between nations, institutions and authors in a particular scientific research field (18). Additionally, the bibliometric approach may easily provide scholars with an overview of the evolution and boundaries of a certain subject of study (19). Several bibliometric studies have looked at the trend in publications in the area of breast cancer or just inflammation (20–22). For instance, a bibliometric study described the relationship between exosomes and breast cancer (23). To date, however, there were no published bibliometric study of the literature relating breast cancer and inflammation. To address this deficiency, we analyzed the scient metric literature in the subject to provide light on the present state of research. Our study aims to provide academics with prospective future research areas by collecting data on yearly publications and citations in connected disciplines, the most productive nations and authors, prominent journals, hot themes, and keyword analysis in the previous decade.

## Methods

### Data acquisition and search strategy

**Figure 1:**
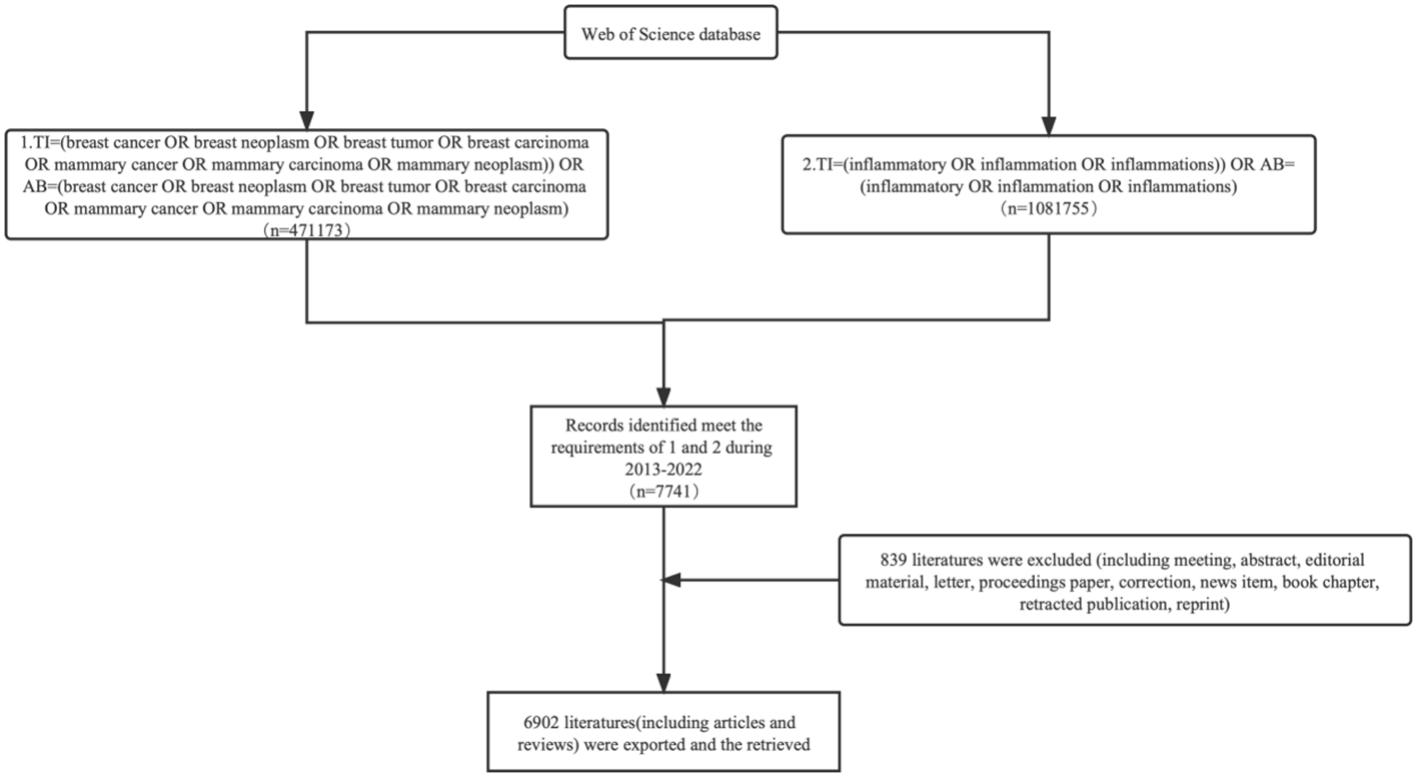
Literature-sifting process flowchart.

Many bibliometricians use the Web of Science Core Collection (WoSCC) database, which includes the Emerging Sources Citation Index (ESCI), the Social Science Citation Index (SSCI), and the Science Citation Index Expanded (SCIE) (24). The following search terms were used: 1. TI = (breast cancer OR breast neoplasm OR breast tumor OR breast carcinoma OR mammary cancer OR mammary carcinoma OR mammary neoplasm)) OR AB = (breast cancer OR breast neoplasm OR breast tumor OR breast carcinoma OR mammary cancer OR mammary carcinoma OR mammary neoplasm) AND 2. TI = (inflammatory OR inflammation OR inflammations)) OR AB= (inflammatory OR inflammation OR inflammations) (25, 26). The records meet the requirements of 1 and 2 during January 1, 2013, to January 1, 2023. 839 irrelevant items, such as meeting abstracts, editorial materials, corrections, letters, retractions, and proceedings papers, were removed from the total of 7741 articles that were retrieved. A total of 6902 pieces of literature were exported, and the ones that were recovered will be exported as complete records and references, saved as plain text files, and kept in the download txt format (**Figure 1**).

### Analysis of data

The Web of Science Core Collection was used to gather all reliable data, which was then imported into CiteSpace, Microsoft Excel 2019, and VOSviewer for analysis. This program was created by Leiden University in the Netherlands to help in the creation, visualization, and exploration of literary data. It is praised for its user-friendly design and distinctive overlay view (27). It may group a collection of strongly connected nodes into different clusters, with the same hue indicating better node correlation (28). Additionally, VOSviewer enables the overlay visualization map, in which nodes’ distance and color depict how they are dispersed throughout two-dimensional spaces (29). In the context of scientometrics and data visualization, CiteSpace is a software program for citation visualization analysis that focuses on the examination of the information that may be found in scientific publications (30). It has an intuitive understanding of the knowledge system’s research hubs, how different areas have evolved, and how to forecast future trends in those subjects. Large-scale data analysis may be done effectively with this technique.

## Results

### The contributions of countries to global publications

**Figure 2:**
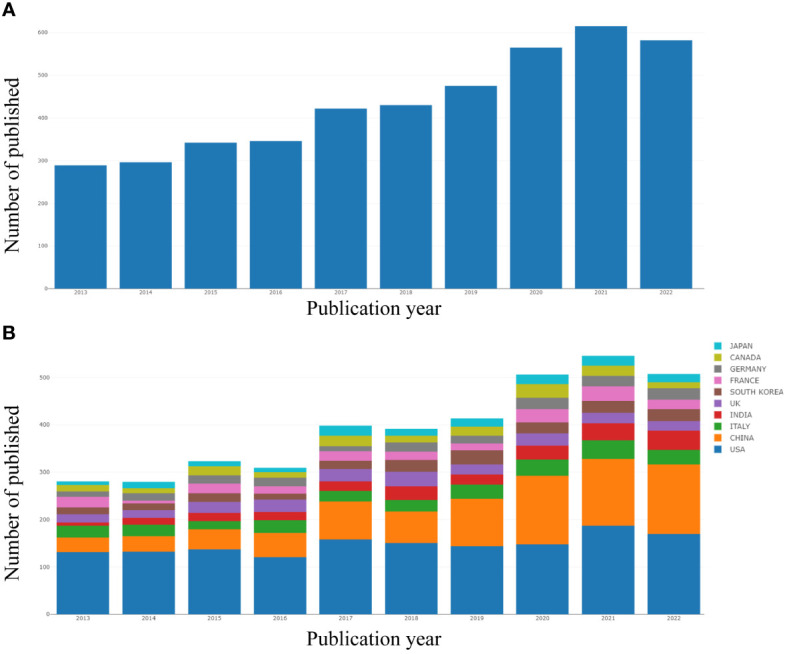
Data on annual publication counts **(A)** and growth rates among the top 10 nations **(B)**.

**Figure 3:**
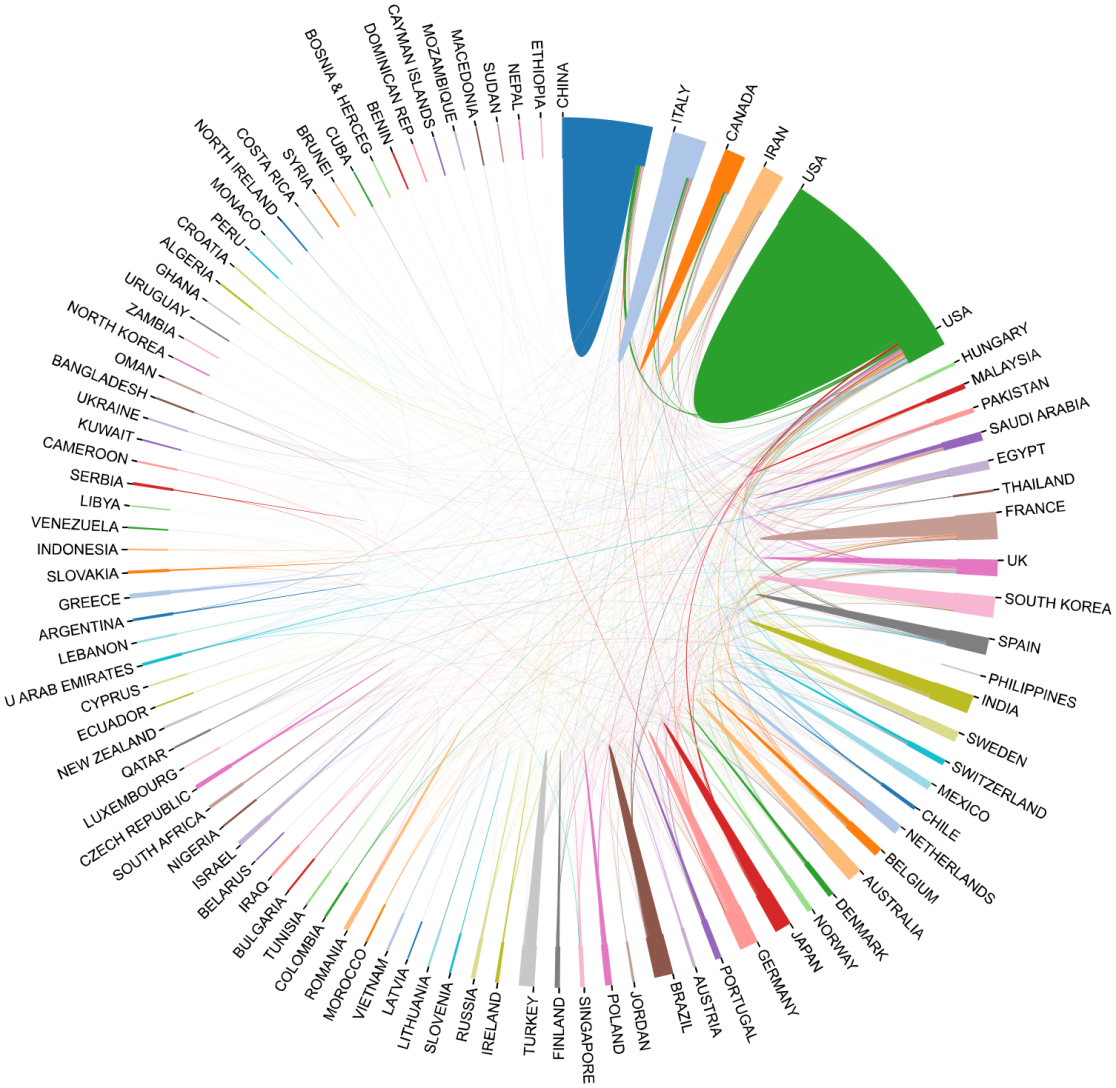
International collaboration in breast cancer inflammation research from 2013 to 2022.

Based on the publications each year, the trend of publishing growth is evaluated. **Figure 2A** shows the yearly trends in article publishing from 2013 to 2022. In general, there was an upward tendency in the number of articles. The most literatures among them were produced in 2021 (n = 988), while the fewest were in 2013 (n = 468). Compared to the prior five years, there have been a lot more publications in the last five years. Research on breast cancer and inflammation is being conducted in at least 120 different nations and areas. The United States has significantly contributed to the research of inflammation in breast cancer during the last ten years, followed by China (1222), Italy (426), India (394), etc (**Figure 2B**; **Table 1**). The study of international collaboration reveals that the United States routinely collaborates with other nations. Although China ranks second in the number of articles published, there is less cooperation with other countries (**Figure 3**).

**Table 1:** The top 10 countries/regions contributing to publications about inflammation in breast cancer.

Rank	Countries	Article counts	Percentage	H-index	Total number of citation	Average number of citations
1	USA	2291	33.19%	105	72675	31.72
2	PEOPLES R CHINA	1222	17.71%	63	22611	18.50
3	ITALY	426	6.17%	52	12518	29.38
4	INDIA	394	5.71%	44	7115	18.06
5	SOUTH KOREA	346	5.01%	46	7689	22.22
6	FRANCE	309	4.48%	41	6506	21.06
7	GERMANY	306	4.43%	48	8768	28.65
8	ENGLAND	293	4.25%	52	11087	37.84
9	CANADA	263	3.81%	42	7326	27.86
10	JAPAN	227	3.29%	37	4805	21.17

### Investigation of the most productive organizations

**Figure 4:**
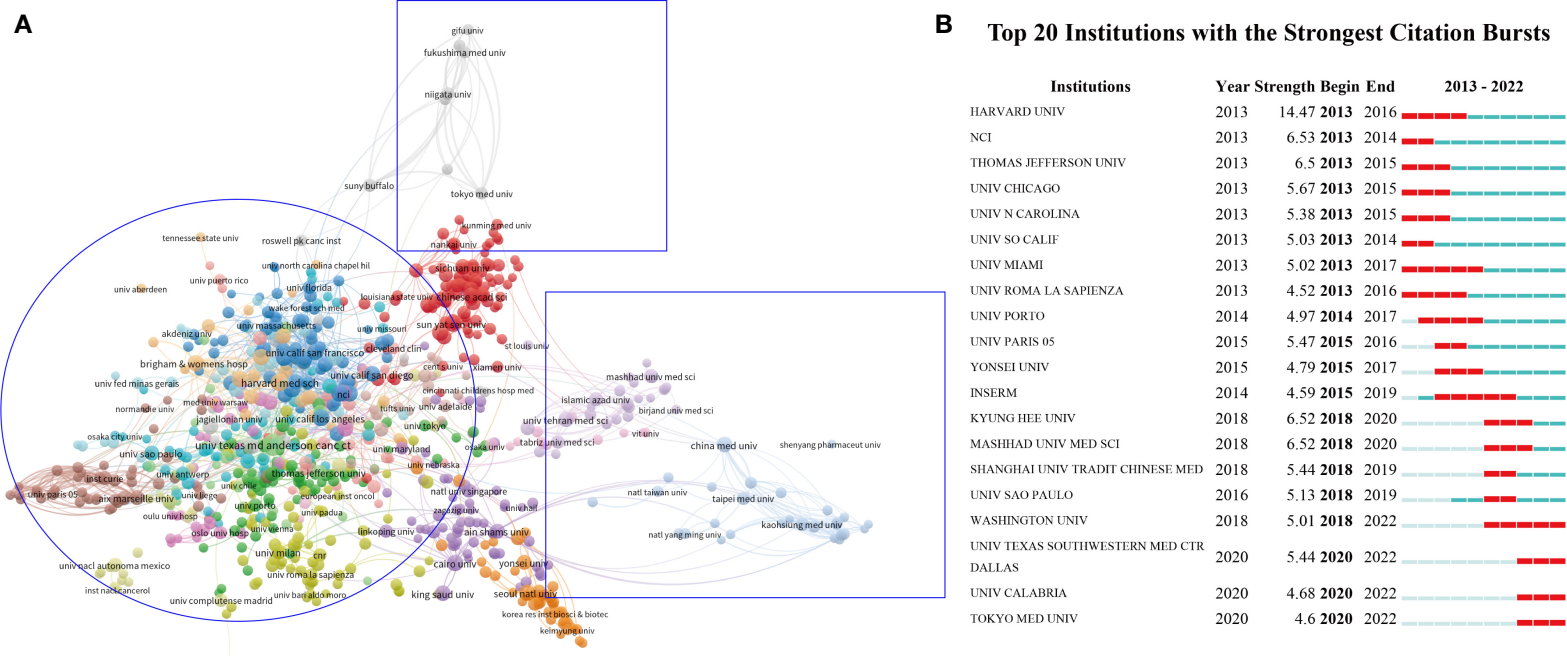
**(A)** The cooperation between different organizations, with blue representing American organizations, red representing Chinese organizations. brown representing France institutions, purple representing the middle east, light yellow representing Britain institutions, yellow representing Italy institutions, grey representing Japan institutions, orange representing Korea institutions and light blue representing rest of Asia institutions. **(B)** CiteSpace visualization map of top 20 institutions with the strongest citation bursts involved in inflammation in breast cancer.

There are more than 6307 institutions studying the role of inflammation in breast cancer. The top 10 universities are shown in **Table 2**. the University of texas system has made the most significant contribution to the study of breast cancer and inflammation with 319 publications and 10025 citations, followed by UT MD Anderson Cancer Center with 245 publications and 8033 citations and University of California System with 223 publications and 7923 citations (**Table 2**). The collaboration between several organizations is shown in **Figure 4A**. We see both regional concentration and general dispersion when we examine the characteristics of the institution partnership network(circles show regional concentration and squares show dispersion in **Figure 4A**). CiteSpace discovered 20 institutions with the strongest citation bursts in **Figure 4B**. The most influential organization from 2013 to 2016 was Harvard University (strength = 14.47). In addition, four institutions including Washington University, University of Texas Southwestern Med Ctr Dallas, University of Calabria and Tokyo Medical University have become hot research centers in recent years.

**Table 2:** The top 10 institutions contributing to publications about inflammation in breast cancer.

Rank	Institutions	Article counts	Percentage	H-index	Total number of citation	Average number of citations
1	UNIVERSITY OF TEXAS SYSTEM	319	4.62%	53	10025	31.43
2	UTMD ANDERSON CANCER CENTER	245	3.55%	48	8033	32.79
3	UNIVERSITY OF CALIFORNIA SYSTEM	223	3.23%	46	7923	35.53
4	HARVARD UNIVERSITY	211	3.06%	47	9754	46.23
5	UDICE FRENCH RESEARCH UNIVERSITIES	174	2.52%	36	4142	23.80
6	EGYPTIAN KNOWLEDGE BANK EKB	171	2.48%	26	2270	13.27
7	INSTITUT NATIONAL DE LA SANTE ET DE LA RECHERCHE MEDICALE INSERM	160	2.32%	32	3374	21.09
8	UNICANCER	154	2.23%	33	3042	19.75
9	HARVARD MEDICAL SCHOOL	124	1.80%	39	6146	49.56
10	NATIONAL INSTITUTES OF HEALTH NIH USA	109	1.58%	32	3529	32.38

### Citation bursts in reference

**Figure 5:**
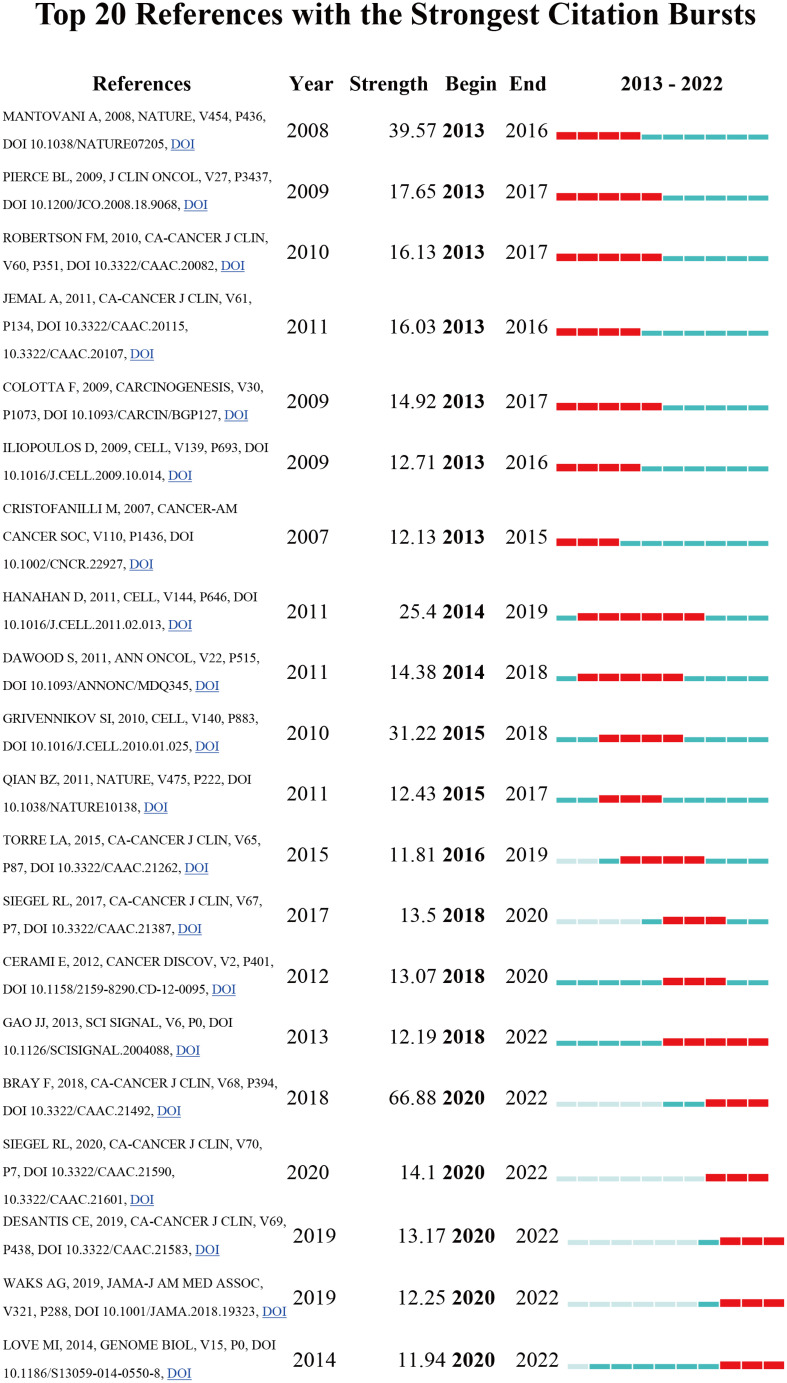
The most often cited top 20 sources. Citations that were particularly high that year are shown in red.

References that get a lot of citations in a short period of time are known as references with citation bursts. CiteSpace detected 20 references in our analysis that had significant bursts of citations (**Figure 5**). The red bar in **Figure 5** denotes significant citation burstiness, and each bar represents a year. “*Global cancer statistics 2018: GLOBOCAN estimates of incidence and death globally for 36 cancers in 185 countries*” was the title of the reference with the highest citation burst (strength = 66.88). It was written by Freddie Bray. Citation spikes between 2020 and 2022. “*Cancer-related inflammation*” written by Alberto Mantovani et al. and published in Nature, had the second-strongest citation burst (strength = 39.57), with bursts occurring from 2013 to 2016. These 20 references had endurance strengths between 2 and 6 years and burst strengths that varied from 11.81 to 66.88 overall. **Table 3** also lists the top 10 studies concerning inflammation in breast cancer that received the most citations from 2013 to 2022.

**Table 3:** The top 10 high-cited papers about inflammation in breast cancer during 2013 to 2022.

Rank	Article Title	Journal	Authors	Publication year	Total citation	Average annual frequency of citations	IF	JCR
1	Macrophage plasticity and polarization in tissue repair and remodelling	JOURNAL OF PATHOLOGY	Mantovani, A; Biswas, SK; (…); Locati, M	2013	1452	145.20	9.883	Q1
2	Genome-wide polygenic scores for common diseases identify individuals with risk equivalent to monogenic mutations	NATURE GENETICS	Khera, AV; Chaffin, M; (…); Kathiresan, S	2018	1168	233.60	41.037	Q1
3	IL-17-producing gamma delta T cells and neutrophils conspire to promote breast cancer metastasis	NATURE	Coffelt, SB; Kersten, K; (…); de Visser, KE	2015	960	120.00	69.504	Q1
4	Iron oxide nanoparticles inhibit tumour growth by inducing pro-inflammatory macrophage polarization in tumour tissues	NATURE NANOTECHNOLOGY	Zanganeh, S; Hutter, G; (…); Daldrup-Link, HE	2016	865	123.57	40.523	Q1
5	The cellular and molecular origin of tumor-associated macrophages	SCIENCE	Franklin, RA; Liao, W; (…); Li, MO	2014	811	90.11	63.714	Q1
6	Subphenotypes in acute respiratory distress syndrome: latent class analysis of data from two randomised controlled trials	LANCET RESPIRATORY MEDICINE	Calfee, CS; Delucchi, K; (…); Matthay, MA	2014	660	73.33	102.642	Q1
7	A Cytoplasmic NF-kappa B Interacting Long Noncoding RNA Blocks I kappa B Phosphorylation and Suppresses Breast Cancer Metastasis	CANCER CELL	Liu, BD; Sun, LJ; (…); Song, EW	2015	657	82.13	38.585	Q1
8	Pertuzumab plus trastuzumab in combination with standard neoadjuvant anthracycline-containing and anthracycline-free chemotherapy regimens in patients with HER2-positive early breast cancer: a randomized phase II cardiac safety study (TRYPHAENA)	ANNALS OF ONCOLOGY	Schneeweiss, A; Chia, S; (…); Cortes, J	2013	651	51.10	51.769	Q1
9	Cyclooxygenase-Dependent Tumor Growth through Evasion of Immunity	CELL	Zelenay, S; van der Veen, AG; (…); Sousa, CRE	2015	616	77.00	66.850	Q1
10	Neutrophils support lung colonization of metastasis-initiating breast cancer cells	NATURE	Wculek, SK and Malanchi, I	2015	608	76.00	69.504	Q1

### The authors’ contributions

**Figure 6:**
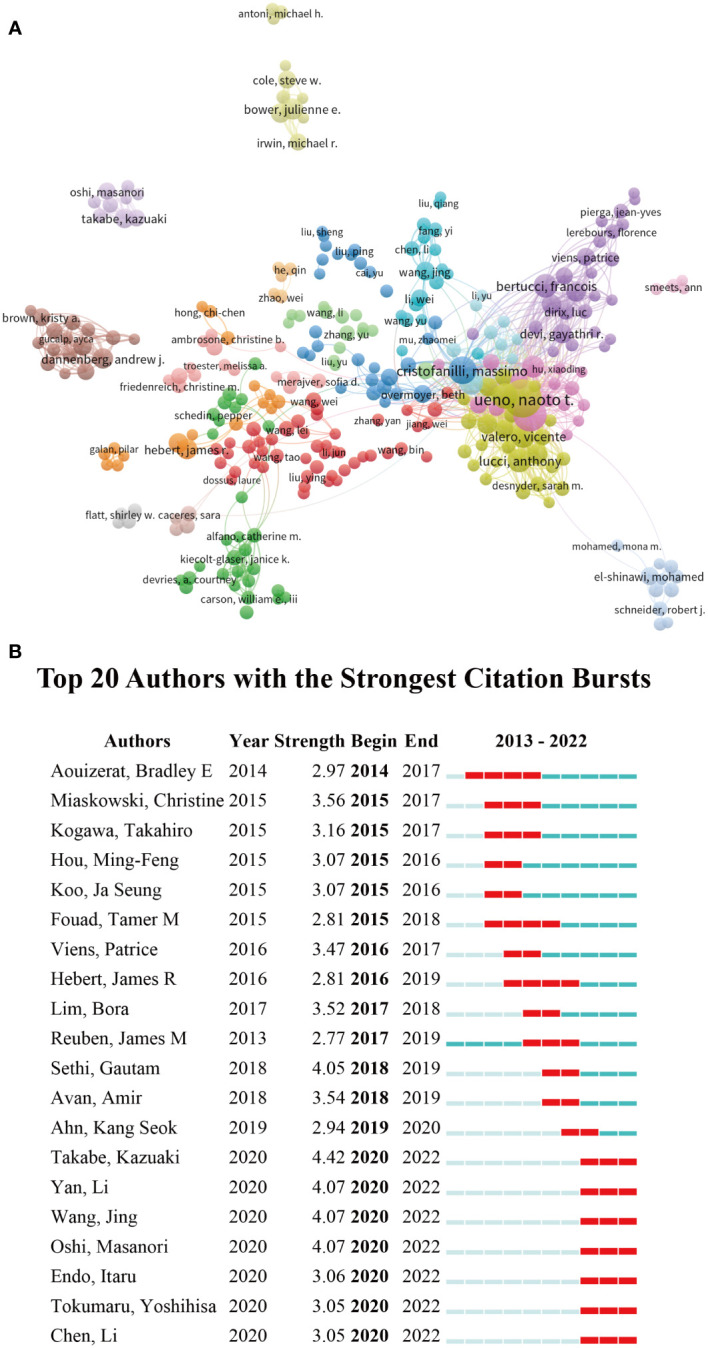
The relationship of research authors of inflammation in breast cancer **(A)**.The 20 most rapidly-cited writers from 2013 to 2022 **(B)**.

**Table 4** is a list of the top 10 writers according to the volume of published works. Ueno NT, a researcher at the MD Anderson Cancer Center at The University of Texas, came in first place among them. He is also at the top of the list in terms of total citations at the same period. Those show that Ueno NT has made outstanding achievements in inflammation and breast cancer researches. We examined the author data using CiteSpace and represented it as a network (**Figure 6A**). The writers were loosely sorted into various colors based on the clustering data. We see both a localized concentration and a general dispersion. CiteSpace found 20 authors in our analysis who had significant bursts of citations (**Figure 6B**). During 2015-2019, the research of Sethi, Gautam and Miaskowski, Christine and Avan, Amir had a great influence. After 2019, the influence of Takabe, Kazuaki and Yan, Li and Wang, Jing gradually increased. And tied for first place (strength=4.07)

**Table 4:** The top 10 most productive authors contributed to publications about inflammation in breast cancer.

Rank	Author	Institution	Article counts	H-index	Total number of citations	Average number of citations
1	Ueno NT	Univ Texas MD Anderson Canc Ctr, Dept Breast Med Oncol, Houston, TX 77030 USA	103	30	2641	25.64
2	Woodward WA	Univ Texas MD Anderson Canc Ctr, Morgan Welch Inflammatory Breast Canc Res Program, Houston, TX 77030 USA	78	30	2178	27.92
3	Cristofanilli M	Fox Chase Canc Ctr, Dept Med Oncol, G Morris Dorrance Jr Endowed Chair Med Oncol, Philadelphia, PA 19111 USA	45	24	1342	29.82
4	Krishnamurthy S	Univ Texas MD Anderson Canc Ctr, Morgan Welch Inflammatory Breast Canc Program & C, Houston, TX 77030 USA	40	19	989	24.73
5	Reuben JM	Univ Texas MD Anderson Canc Ctr, Morgan Welch Inflammatory Breast Canc Res Program, Houston, TX 77030 USA	39	25	1496	38.36
6	Bertucci F	Inst Paoli Calmettes, Dept Med Oncol & Mol Oncol, Marseille, France	36	17	910	25.28
7	Valero V	Univ Texas MD Anderson Canc Ctr, Houston, TX 77030 USA	32	16	819	25.59
8	Lucci A	Univ Texas MD Anderson Canc Ctr, Morgan Welch Inflammatory Breast Canc Program & C, Houston, TX 77030 USA	31	14	692	22.32
9	Debeb BG	Univ Texas MD Anderson Canc Ctr, Dept Breast Med Oncol, Houston, TX 77030 USA	26	14	602	23.15
10	Bower JE	Univ Calif Los Angeles, Dept Psychol, Los Angeles, CA 90095 USA	25	14	991	39.64

### Research published in journals

1579 journals have published articles in the last ten years on inflammation and breast cancer. Of the 6902 articles field of inflammation in breast cancer we collected, 1104 (15.99%) of the top 10 journals’ publications (**Table 5**). CANCERS (IF = 6.575), PLOS ONE (IF = 3.752), and INTERNATIONAL JOURNAL OF MOLECULAR SCIENCES (IF = 6.208) are the top 3 publications in terms of quantity. The top 3 journals in terms of average citation count are PLOS ONE (IF = 3.752), ONCOTARGET (IF = 4.345), and BREAST CANCER RESEARCH (IF = 8.408). The three journals are classified as belonging to Q1 and Q2 by JCR of 2022.

**Table 5:** The top 10 most active journals that published articles about inflammation in breast cancer.

Rank	Journal title	Article counts	Percentage	H-index	Total number of citations	Average number of citations	IF	JCR
1	CANCERS	175	2.54%	23	1906	10.89	6.575	Q1
2	PLOS ONE	149	2.16%	34	3474	23.32	3.752	Q2
3	INTERNATIONAL JOURNAL OF MOLECULAR SCIENCES	137	1.99%	26	2257	16.47	6.208	Q1
4	SCIENTIFIC REPORTS	113	1.64%	27	2578	22.81	4.996	Q2
5	ONCOTARGET	106	1.54%	33	3273	30.88	4.345	Q2
6	BREAST CANCER RESEARCH AND TREATMENT	104	1.51%	28	2420	23.27	4.624	Q2
7	FRONTIERS IN ONCOLOGY	96	1.39%	15	890	9.27	5.738	Q2
8	BMC CANCER	82	1.19%	26	1715	20.91	4.638	Q2
9	MOLECULES	72	1.04%	19	1348	18.72	4.927	Q2
10	BREAST CANCER RESEARCH	70	1.01%	30	2212	31.60	8.408	Q1

### Analysis of keywords in publications on breast cancer and inflammation

**Figure 7:**
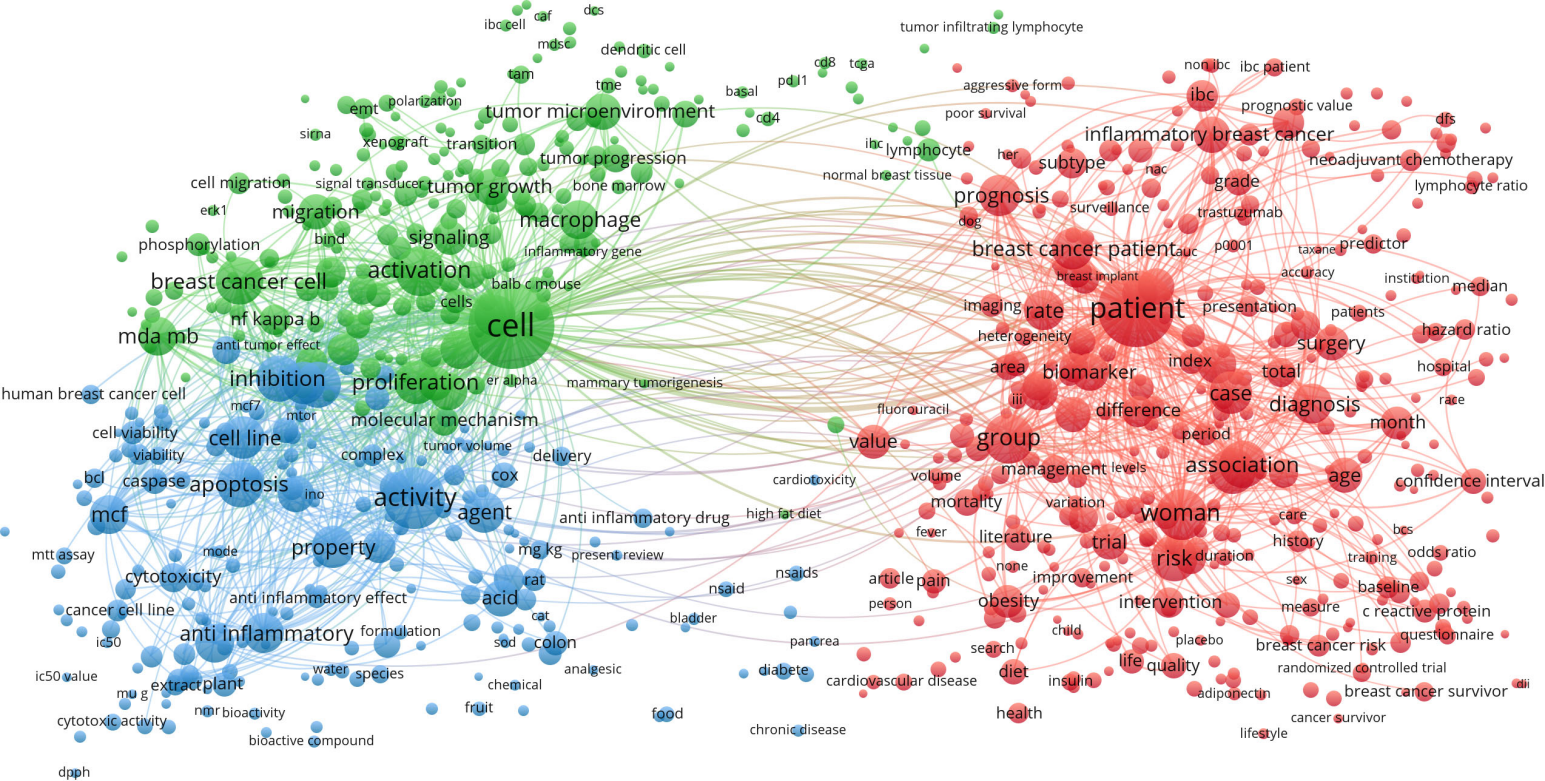
Analyzing the role of inflammation in breast cancer research using a co-word network and clustering of relevant keywords. Co-word frequency is represented by node size, co-word intensity by connection thickness, co-word network clustering results by color, and a tighter association between two words is shown by a node’s proximity to another node of the same color.

We used VOSviewer to do cluster analysis after filtering out terms with more than 10 occurrences (**Figure 7**). The connections between the keywords are stronger when the lines connecting the nodes are thicker.

**Figure 8:**
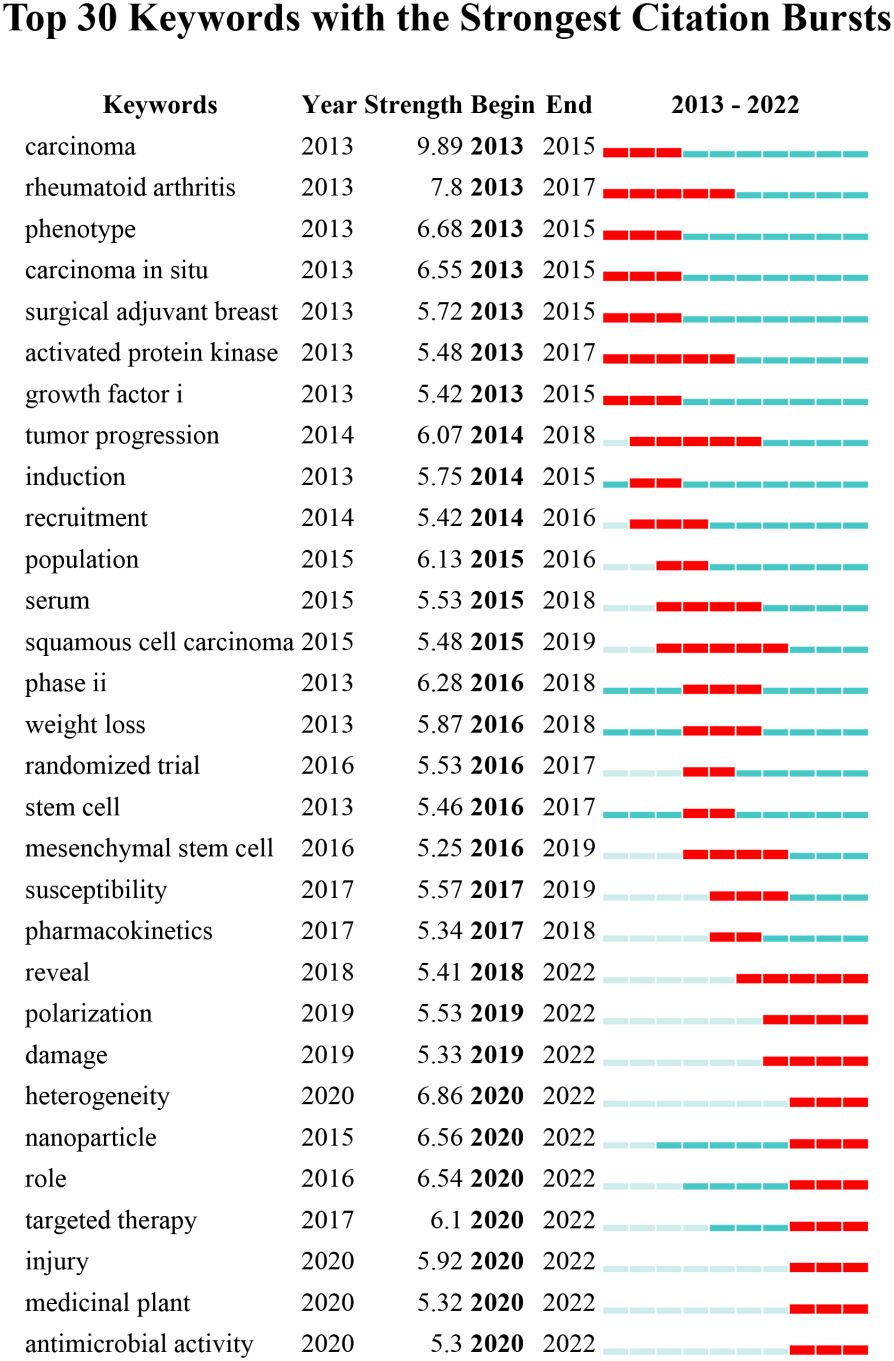
In this visualization of CiteSpace’s analysis of the top 30 keyword explosions, “strong” denotes the magnitude of the explosion, “begin” shows the year in which the explosion began, and “end” indicates the year in which the explosion ended; a red dotted line depicts the explosion’s length. The full time frame, from 2013 to 2022, is shown by the blue line.

As shown in **Figure 7**, VOSviewer assessed and grouped the whole keywords that were taken from the title and abstract of 6902 relevant articles. 2467 keywords were categorized into three mechanism-associated subclusters by viewing keywords with high co-occurrences, which were defined as items that appeared more than 10 times: “patient” (red), “cell” (green), and “anti-inflammatory” (blue). Relevant terms within the red cluster included enhanced group, surgery, biomarker, and prognosis. In the cluster of green, the main keywords were activation, tumor growth, cell proliferation, migration, tumor microenvironment and macrophage. Relevant terms within the blue cluster included inhibition, apoptosis, activity, and anti-inflammatory medicine. The trend topic analysis of the keywords revealed that from 2013 to 2017 (**Figure 8**), microvesicles and microparticles were the primary study topics, while tumor progression, activated protein kinase, phenotypic, and rheumatoid arthritis were the top keywords. Mesenchymal stem cells, pharmacokinetics, and other significant terms have emerged as researchers actively investigate the pathophysiology and therapeutic potential of stem cells and drugs in breast cancer since 2017. Additionally, the following five keywords—heterogeneity, nanoparticle, targeted therapy, medicinal plant, and antimicrobial activity—appeared frequently in the previous three years (2020–2022), so they are very likely to represent the hottest areas of research on inflammation in breast cancer at the moment (**Figure 8**).

## Discussion

So far as we are aware, this is the first bibliometric investigation of the possible connection between inflammation and breast cancer. 6902 papers were included in the analysis. There was an increasing tendency in publications. There were 36191 writers from 6307 institutions and 120 countries involved in the development of this field. Among the top writers, NT Ueno had 103 papers, received 2641 citations, and had an h-index of 30; WA Woodward had 78 papers, received 2178 citations, and had an h-index of 30; and MC Cristofanilli had 45 papers, received 1342 citations, and had an h-index of 24. With 319, 245, and 223 publications respectively, The University of Texas System, MD Anderson Cancer Center, and the University of California, respectively, were the top three universities. The United States produced 2291 papers, China produced 1222 documents, and Italy produced 426 records, respectively. There were a grand total of 1579 periodicals that advanced this study. CANCERS topped the list with 175 articles, followed by PLOS ONE with 149 articles, and the INTERNATIONAL JOURNAL OF MOLECULAR SCIENCES with 137 articles. The clusters are divided into three distinct lines of inquiry. There are three research directions in the clusters. The green cluster includes the important keywords of cell, tumor microenvironment, cell proliferation, signalling, etc. The blue cluster includes the important keywords of apoptosis, inhibition, activity and anti-inflammatory. The red cluster includes the important keywords of patients, biomaker, prognosis and surgery.

### Areas of advancement and hotspots

Based on clustering research and keywords, the progress field of research hotspots between inflammation and breast cancer are found as follows: Specifically, the red cluster draws attention to the importance of clinical research and breast cancer patients. Staging of the tumor and lymph nodes is used to determine the prognosis and treatment choices for breast cancer. Factors include lymph node involvement, histologic grade, hormone receptor status, HER2 overexpression, comorbidities, menopausal status, and patient age are also significant (31). Breast cancer treatment options often consist of radical mastectomy, radiation therapy, chemotherapy, and endocrine therapy. Historically, the gold standard for treating invasive breast cancer in its early stages has been a procedure called a “modified radical mastectomy.” But breast-conserving surgery has gained popularity in recent years. With this treatment, the patient’s breast is left looking more natural than it would after a radical mastectomy since just the cancerous tissue is removed. Local recurrence is reduced and cancer-specific survival rates are increased to levels comparable to those with mastectomy when radiation treatment is administered after breast-conserving surgery (32). Whether or not radiation treatment and adjuvant systemic therapy are required is dependent on the status of axillary lymph nodes (ALNs). The disease-free and overall survival of patients with early-stage breast cancer who have a negative sentinel lymph node are significantly higher compared to those who have a negative axillary lymph nodes dissection, according to a prospective investigation. Following breast-conserving surgery, subclinical illness is often treated with whole-breast radiotherapy. A meta-analysis of 10 RCTs comparing breast-conserving surgery with and without radiation revealed that the use of radiation substantially decreased the five-year local recurrence rate. The use of chemotherapy, hormone treatment, and tissue targeted medicines in conjunction with definitive local therapy (surgery, radiation therapy, or both) significantly reduces cancer recurrence and disease-specific mortality. In addition, molecular diagnostics, molecular resection, and targeted therapeutics provide the possibility to personalize breast cancer therapy for each individual patient.

Cluster 2 in green color mainly highlights keywords on the mechanism of tumor growth such as cell proliferation, tumor progression, tumor growth and tumor microenvironment. Cluster 3 in blue mainly highlights regulation cell activity, cell death and inflammation for the treatment of breast cancer. Clusters 2 and 3 describe the area of basic research in breast cancer. As an important keyword, inflammation has many associations with other keywords in clusters 2 and 3.

Inflammation is involved in epithelial-mesenchymal transition, epigenetic control, cellular plasticity and intra-tumoral heterogeneity, which is contributed to the progression of breast cancer (33–35). The tumor microenvironment (TME) also plays an important role in the progression of breast cancer. Inflammation can increase the risk of cancer promoting cells by infiltrating the the tumor microenvironment, by releasing cytokines, growth factors, chemokines, and proangiogenic factors (36–38). Breast tumor microenvironment is dominated by macrophages and fibroblasts. During inflammatory reactions, fibroblasts and mesenchymal stem cells are often brought in to help (39). It is noteworthy that cancer-associated fibroblasts (CAFs), the main stromal cells that contribute to the TME in breast cancer, were examined at the single cell level and subsequently classified into many subclasses with various functional programs and prognostic significance (40–42). TAMs (tumor-associated macrophages) release cytokines, chemokines, and enzymes that promote angiogenesis, tumor growth, and cell proliferation (43). Additionally, by collaborating with immune and epithelial cells to control the cellular environment *via* the release of cytokines and chemokines, macrophages contribute to both innate and adaptive immunity (44). Many different kinds of cells in the TME secrete cytokines, which then act either locally (through autocrine and paracrine) or systemically *via* direct interactions with their respective membrane receptors (45). Multiple aspects of tumor immunity include members of the interleukin-1 (IL-1) family (46). There are seven pro-inflammatory ligands and four anti-inflammatory cytokines, all playing important roles in host defense and the inflammatory processes that may lead to cancer (47, 48). Collectively, deep insight into mechanisms of tumorigenesis and development from the perspective of inflammation is indeed essential to explore the novel therapeutic mechanism underlying breast cancer.

### Strengths and limitations

There are several advantages to this research. To begin, for the first time, we conducted a systematic bibliometric analysis of inflammation in breast cancer, which may give complete recommendations for researchers that focus on related research. Second, we employed two bibliometric tools (VOSviewer and CiteSpace) which are extensively used in the area of bibliometrics. Therefore, it is probable that our method of analyzing the data will be objective. Finally, compared with traditional reviews, bibliometric analysis can provide a more comprehensive insight into hot spots and frontiers. However, it is important to acknowledge numerous limitations. To begin, we only choose to publication in English, which may lead to selection bias. Second, we only choose the articles in the WOSCC database, it will inevitably result in bibliography omissions. Third, citation metrics are time-dependent, meaning that recent articles are likely to be cited less often than previous articles, mainly due to the date of publication. As a result, bibliometrics may be biased in selecting the most popular articles. It is possible that some recently released high-quality articles will briefly be ranked lower than classic publications.

## Conclusion

This is the first bibliometric study to evaluate the quantity and quality of information available in the literature on breast cancer and inflammation research. The increasing volume of yearly publications suggests that this research field has gained more attention. In addition, the United States having the largest number of publications. This study has mapped the worldwide landscape of researchers and institutions studying the relationship between breast cancer and inflammation. The highest output was found in CANCERS, while the highest IF was found in BREAST CANCER RESEARCH among the top 10 journals. In addition, the focus of breast cancer study gradually shifts from phenotype study to therapeutic research. It is recommended to pay attention to the latest hot spots, such as targeted therapy,antimicrobial activity and nanoparticle. Meanwhile, many researchers believe that inflammatory cytokines, cell death, and anti-inflammation are the most current and important topics in the field. Those just entering the field of study or those tasked with formulating policy can benefit greatly from these results.

## Data availability statement

The original contributions presented in the study are included in the article/supplementary material. Further inquiries can be directed to the corresponding authors.

## Author contributions

GM conceived the study and wrote the article, HX and SY helped perform the data and statistical analysis. FC and WW helped perform the literature search and data acquisition. GZ and FH revised this manuscript critically for important intellectual content. YG revised the article. GM, HX, SY and FC have contributed equally to this work. All authors contributed to the article and approved the submitted version.
